# Ameliorative Effects of Peptides Derived from Oyster (*Crassostrea gigas*) on Immunomodulatory Function and Gut Microbiota Structure in Cyclophosphamide-Treated Mice

**DOI:** 10.3390/md19080456

**Published:** 2021-08-11

**Authors:** Xing-Wei Xiang, Hui-Zhen Zheng, Rui Wang, Hui Chen, Jin-Xing Xiao, Bin Zheng, Shu-Lai Liu, Yu-Ting Ding

**Affiliations:** 1 College of Food Science and Technology, Zhejiang University of Technology, Hangzhou 310014, China; xxw11086@zjut.edu.cn (X.-W.X.); 2111926028@zjut.edu.cn (H.-Z.Z.); 2111926027@zjut.edu.cn (R.W.); chenhui2020@zjut.edu.cn (H.C.); slliu@zjut.edu.cn (S.-L.L.); 2 Key Laboratory of Marine Fishery Resources Exploitment & Utilization of Zhejiang Province, Hangzhou 310014, China; 3 Ocean Research Center of Zhoushan, Zhejiang University, Zhoushan 316000, China; 4 Food and Pharmacy College, Zhejiang Ocean University, Zhoushan 316000, China; zhengb@zjou.edu.cn

**Keywords:** oyster (*Crassostrea gigas*), peptides, cyclophosphamide, immunomodulatory, gut microbiota, short-chain fatty acid

## Abstract

The intestinal flora is recognized as a significant contributor to the immune system. In this research, the protective effects of oyster peptides on immune regulation and intestinal microbiota were investigated in mice treated with cyclophosphamide. The results showed that oyster peptides restored the indexes of thymus, spleen and liver, stimulated cytokines secretion and promoted the relative mRNA levels of Th1/Th2 cytokines (IL-2, IFN-γ, IL-4 and IL-10). The mRNA levels of Occludin, Claudin-1, ZO-1, and Mucin-2 were up-regulated, and the NF-κB signaling pathway was also activated after oyster peptides administration. Furthermore, oyster peptides treatment reduced the proportion of *Firmicutes*/*Bacteroidetes*, increased the relative abundance of *Alistipes*, *Lactobacillus*, *Rikenell* and the content of short-chain fatty acids, and reversed the composition of intestinal microflora similar to that of normal mice. In conclusion, oyster peptides effectively ameliorated cyclophosphamide-induced intestinal damage and modified gut microbiota structure in mice, and might be utilized as a beneficial ingredient in functional foods for immune regulation.

## 1. Introduction

Currently, autoimmune diseases and cancer are global health problems [1,2]. Cyclophosphamide (Cy) is widely used to treat a variety of cancers and autoimmune diseases while immunosuppression is one of the dominating adverse reactions [3]. Cy is often used to induce immunosuppression in mice. The use of large doses of Cy will lead to many side effects, such as disruption of the intestinal barrier, increased intestinal permeability, damage to the gastrointestinal mucosa, increased exposure of immunodeficiency and secondary infections, disruption of the intestinal flora and so on [4,5]. Hence, to reduce the side effects of Cy, the research and development of immune modulators are of great importance. Natural products are abundant in source, safe and have low toxic side effects, which have been widely developed to enhance immunity [6,7].

The immune system is a highly complex defense mechanism that protects against homeostasis disturbances caused by pathogens, injuries, external pollutants, and infections [8]. When the immune function of the human body is suppressed, it will fatally be attacked by infections, cancer and other associated diseases [9]. There are mainly four forms of T helper cells: type I helper T cells (Th1), type II helper T cells (Th2), T helper cell 17 (Th17), and regulatory T (Treg) cells. Th1 and Th2 are two subsets of Th cells, which play a key role in the regulation of adaptive immunity [10,11]. Th1 and Th2 cells are in dynamic equilibrium in healthy hosts. The destruction of the Th1/Th2 balance is involved in a variety of immune diseases. Interferon-γ (IFN-γ) and tumor necrosis factor α (TNF-α) are characteristic cytokines of Th1 cells, which are considered to be key to cell-mediated inflammation. Th2 cells mainly produce interleukin (IL)-4, IL-5, IL-13, and protect the host against extracellular pathogens [12]. They act primarily on epithelial tissue, especially in the lungs and intestines [13]. In addition, IL-10 was mostly related to the inhibitory function of Treg cells, which were regarded as crucial immunoregulatory factors in numerous autoimmune diseases and inflammation [14,15]. It is of great significance to maintain the body’s immune system well-balanced and enhance the immunity for the prevention and treatment of the occurrence of immunity-related diseases. Collagen hydrolysates from yak (*Bos grunniens*) bone have been reported to increase the level of inflammatory cytokines in the serum of immunodeficient mice, thereby improving the immune function of the mice [16].

The gut is the largest digestive and absorption organ, and also an effective defense barrier. The healthy mucosal barrier should protect the host from disease-causing toxins and microorganisms while performing the function of absorption [14,17]. The gut microbiome is a complex ecosystem, consisting of trillions of microorganisms, which perform a range of physiological functions in the gastrointestinal tract of the host [18]. The host mucosal immune network system includes innate immunity and acquired immunity. The innate immune system promotes the growth of beneficial flora to a certain extent, so as to maintain a stable community environment in the intestine [19]. The mucosal immune system is able to respond to potentially pathogenic microorganisms, eliminate invasive pathogens, and keep a status of tolerance to a variety of beneficial symbiotic bacteria [20,21]. In addition, the gut microbiome is critical to the homeostasis of the intestinal barrier. In recent years, it has been found that the interaction between intestinal microorganisms and host metabolism could regulate host immunity, which has attracted widespread attention [22,23]. The intestinal microbiota is the basis for the development of the host immune system, which participates in the maintenance of intestinal homeostasis by stimulating the immune response [24,25]. Cy could lead to the destruction of intestinal microbiota structure and increase the number of pathogenic bacteria in the intestinal microbiota [4]. Many immune-related diseases in the host, such as cancer, viral influenza, and obesity, are caused by an imbalance in the gut microbiota, which is complex and influenced by internal and external factors. In addition to helping digestion, the gut microbiome also produces short-chain fatty acids (SCFAs), which regulate immune responses in a timely manner and eliminate inflammation [26,27].

In recent years, the development of high value-added food by using proteolytic compounds or bioactive peptides has attracted more and more attention worldwide [28]. Bioactive peptides are peptide compounds extracted from proteins, which could regulate the life activities of organisms, including improving immunity, regulating hormones, lowering blood lipids, having antibacterial and antiviral properties, etc. [29]. Recent research studies have shown that food-source polypeptides have multivarious regulatory effects on immune responses, including regulation of the production of cytokines and antibodies, stimulating lymphocyte proliferation, enhancing the phagocytic capacity of macrophages, enhancing the activity of killer cells, and improving the body’s ability to resist pathogen invasion and inhibiting host pro-inflammatory response to lipopolysaccharide (LPS) [30,31]. For example, whey proteolytic hydrolysates have immune-stimulating effects on splenocyte proliferation and secretion of IL-2 and IFN-γ [32]. Pan et al. concluded that milk proteolytic compounds enhanced the immunity of mice via increasing the weight of the immune organs and stimulating macrophage phagocytosis [33]. Immunoregulatory peptides have been discovered from several protein sources, including eggs [34], clams [35], shellfish [36], oysters [37,38], soybeans [39], etc. However, with the rapid growth of the world population and the overuse of land resources, marine organisms have been considered as potential sources of bioactive peptides [40]. Among the numerous bioactive peptides, the number of peptides from the ocean is very large. It has been reported that the protein hydrolysates obtained from roe by Chalamaiah et al. [31] have immune-protective effects on both innate and adaptive immunity in mice. In addition, shark-derived protein hydrolysates protected the intestinal barrier of immunosuppressed mice by up-regulating the cytokines production [41].

Oyster (*Crassostrea gigas*) is a marine bivalve mollusk widely distributed in coastal areas [42]. Due to its high commercial value, its cultivation contributes significantly to the development of aquaculture and the marine economy in China. Oyster possesses a high nutrient value, because of its abundant microelements, energetic polypeptides, amino acids and other nutrient fractions, which can relieve mental anxiety, promote blood circulation and remove blood stasis. It has been reported that oyster protein hydrolysate played an immune protective role in BALB/c mice by enhancing spleen lymphocyte proliferation and macrophage phagocytosis etc. [37,38,43]. However, the regulating effect of oyster peptides (OP) on intestinal mucosal immunity and intestinal microflora in immunosuppression mice remains to be studied. In this study, Cy was used to create an immunosuppression model, and the immunomodulatory effect of OP was verified by detecting inflammatory factors, intestinal permeability, related cell pathways, intestinal microflora structure and other immune indicators after OP administration. The results of this study provide experimental evidence for using OP as a natural immune modulator to prevent inflammation-related intestinal diseases. 

## 2. Results

### 2.1. Effects of OP on the Immune Organ Indices

**Figure 1:**
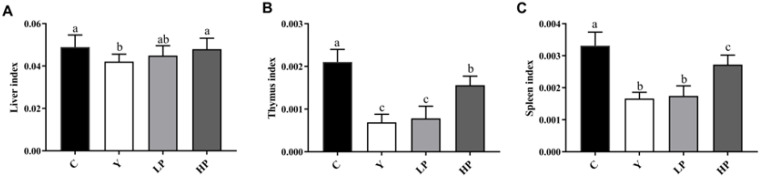
The effect of oyster peptides (OP) on the immune organ index. (**A**) Liver index (**B**) Thymus Index (**C**) Spleen index. Data are presented as the mean ± SD (n = 6). Bars with different letters are significantly different (*p* < 0.05). C: the control group; Y: the cyclophosphamide(Cy)-treated group; LP: Cy + 200 mg/kg OP; HP: Cy + 400 mg/kg OP.

The changes in the liver, thymus and spleen index can directly reflect the immune function in the body. Compared with the C group, the liver, thymus, and spleen index were significantly reduced in the Y group (Figure 1). After the intake of OP, the spleen, thymus and liver index in the HP group were significantly higher than that of the Y group (*p* < 0.05), but there was no statistically significant difference between the Y group and the LP group. The results showed that a high dose of OP can effectively increase the immune organ index of mice (*p* < 0.05), suggesting that OP can reverse the immune organ atrophy induced by Cy.

### 2.2. Effects of OP on Intestinal Barrier

**Figure 2:**
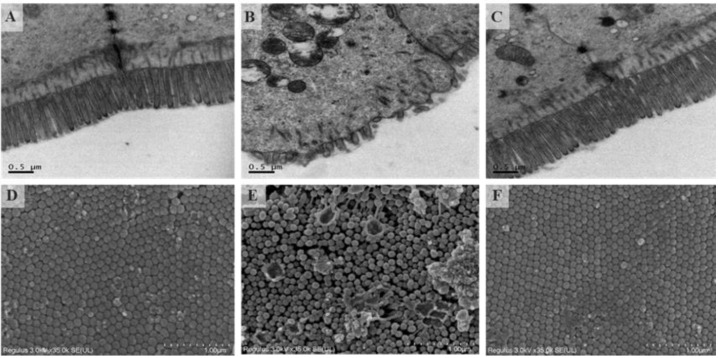
Effects of OP on the epithelial ultrastructure in mice. (TEM, 20 K×, (**A**): the control group; (**B**): the Cy-treated group; (**C**): Cy + 400 mg/kg OP); Effects of OP on the villi of ileum in mice (SEM, 35 K×, (**D**):the control group; (**E**): the Cy-treated group; (**F**): Cy + 400 mg/kg OP).

Transmission electron micrograph of the ileum of HP (400 mg OP/kg BW/day) treated mice are shown in Figure 2A–C. The microvilli villi in group C were neatly arranged, long and dense, almost without gaps, and tight connections were clearly visible. Compared with the C group, intestinal microvilli in the Y group were found to be rare and disorderly arranged, and their tight connections were less regular. There was a great improvement in the HP group. Scanning electron micrograph of ileum is shown in Figure 2D–F. The height of the microvilli in the Y group was inconsistent and the arrangement was irregular. Some microvilli were damaged and collapsed. The microvilli of the C group and HP group were relatively flat, with small gaps and smooth surfaces. The results suggested that OP can repair the ileum damage caused by Cy in mice.

**Figure 3:**
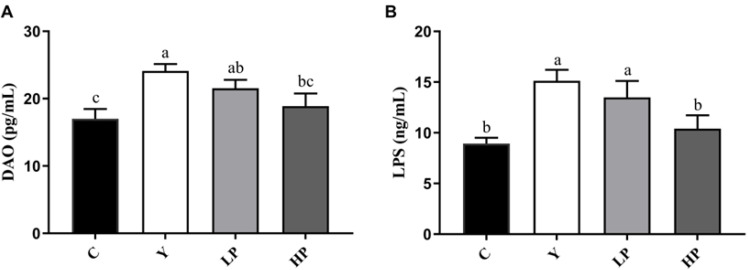
Effects of OP on the contents of (**A**) DAO and (**B**) LPS in serum of mice. Data are presented as the mean ± SD (n = 3). Different letters indicate significant (*p* < 0.05) differences. C: the control group; Y: the Cy-treated group; LP: Cy + 200 mg/kg OP; HP: Cy + 400 mg/kg OP.

Cy not only destroyed the intestinal barrier, but also altered intestinal permeability. In Figure 3, compared with the C group, the levels of DAO and LPS in the Y group were increased significantly (*p* < 0.05). After administration of OP, the levels of DAO and LPS were significantly lower in the LP and HP groups than that in the Y group (*p* < 0.05), in a dose-dependent manner.

### 2.3. Effects of OP on the sIgA Secretion in Ileum

**Figure 4:**
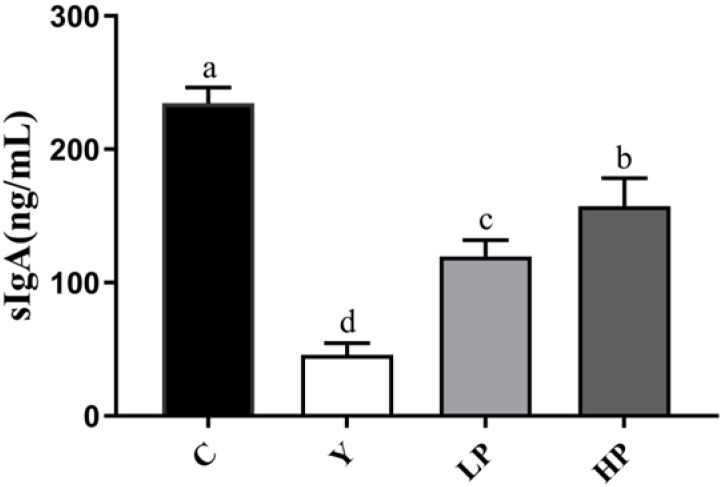
Effects of OP on the contents of sIgA in the ileum of mice. Data are presented as the mean ± SD (n = 3). Different letters indicate significant (*p* < 0.05) differences. C: the control group; Y: the Cy-treated group; LP: Cy + 200 mg/kg OP; HP: Cy + 400 mg/kg OP.

As shown in Figure 4, after Cy treatment, the sIgA content was significantly lower in the Y group (*p* < 0.05) than that in the C group, while those in LP and HP group significantly increased (*p* < 0.05). The results suggested that OP could promote intestinal mucosal immunity to some extent.

### 2.4. Effects of OP on Cytokines Production in Ileum

**Figure 5:**
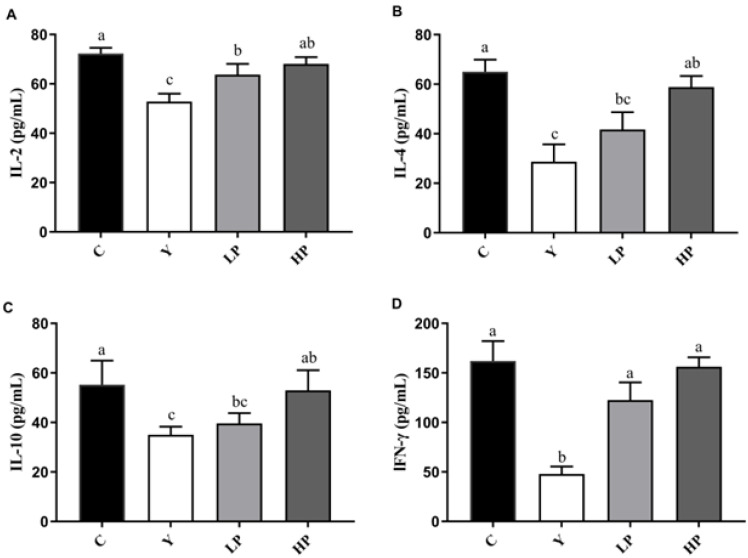
Effects of OP on the contents of (**A**) IL-2, (**B**) IL-4, (**C**) IL-10 and (**D**) IFN-γ in the ileum of mice. Data are presented as the mean ± SD (n = 3). Different letters indicate significant (*p* < 0.05) differences. C: the control group; Y: the Cy-treated group; LP: Cy + 200 mg/kg OP; HP: Cy + 400 mg/kg OP.

The levels of inflammatory factors (IL-2, IFN-γ, IL-4 and IL-10) were detected by ELISA kits to further explain the immunomodulatory effect of OP on the intestinal mucosa, as shown in Figure 5A–D. Compared with the C group, the secretion of IL-2, IFN-γ, IL-4 and IL-10 in the Y group was significantly decreased (*p* < 0.05). OP significantly enhanced the contents of IL-2, IFN-γ, IL-4 and IL-10 in the ileum of immunosuppressed mice in a dose-dependent manner (*p* < 0.05). Therefore, our results indicated that OP promotes the secretion of Th1 and Th2 cytokines and had an immune protective effect on the ileum of immunosuppression mice.

### 2.5. Effects of OP on the Relative mRNA Expression of Ileum in Cy-Treated Mice

**Figure 6:**
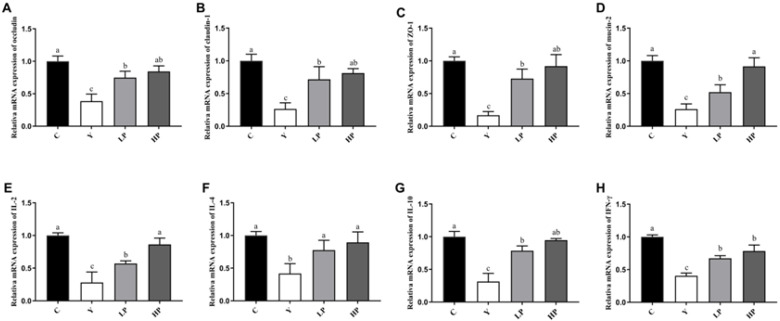
Effects of OP on the relative expression of (**A**) occludin, (**B**) claudin-1 (**C**) ZO-1, (**D**) mucin-2 and (**E**) IL-2, (**F**) IL-4, (**G**) IL-10, (**H**) IFN-γ in the ileum of mice. Data are presented as the mean ± SD (n = 3). Different letters indicate significant (*p* < 0.05) differences. C: the control group; Y: the Cy-treated group; LP: Cy + 200 mg/kg OP; HP: Cy + 400 mg/kg OP.

The effects of OP on the relative gene levels of intestinal tight junction and inflammatory cytokines were determined by RT-PCR. As shown in Figure 6A–D, the relative mRNA expressions of Occludin, Claudin-1 and ZO-1 in the Y group were substantially decreased (*p* < 0.05), suggesting that Cy destroyed the tight junctions between epithelial cells. However, after administration of OP, the mRNA levels of Occludin, Claudin-1 and ZO-1 were significantly enhanced (*p* < 0.05), in a dose-dependent manner. Compared with the C group, the mRNA level of mucin-2 in the Y group was significantly decreased (*p* < 0.05), and this situation was significantly improved in LP and HP groups. Mucin-2 is a kind of glycoprotein secreted by epithelial cells, which plays a role in cell protection and lubrication in tissue epithelium [10]. The results indicated that OP has a protective effect on the intestinal tract.

Moreover, in Figure 6E–H, the relative mRNA levels of IL-2, IFN-γ, IL-4 and IL-10 of ileum in the Y group were decreased significantly (*p* < 0.05), indicating that the treatment with Cy resulted in ileum inflammation. After the administration of OP, the mRNA levels of IL-2, IFN-γ, IL-4 and IL-10 in the LP and HP groups increased significantly (*p* < 0.05), and there was a positive correlation with OP dose. The results suggested that OP had a relieving effect on Cy-induced ileum inflammation.

### 2.6. OP Regulated NF-κB Pathway Key Proteins

**Figure 7:**
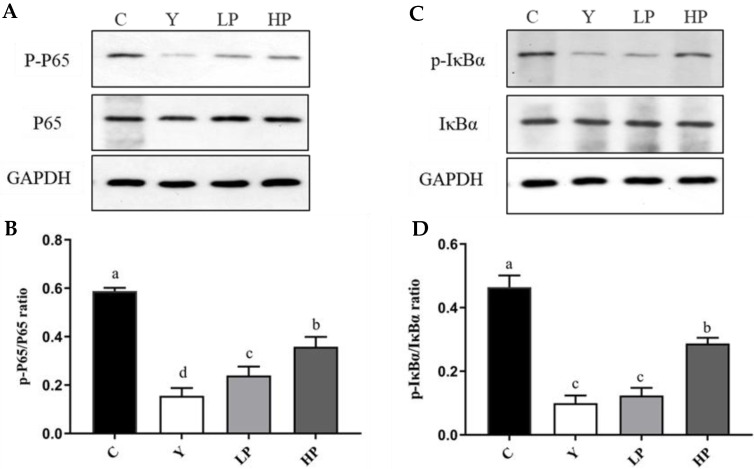
Effects of OP on the NF-κB pathway in the ileum of Cy-treated mice. (**A**) The protein expression levels of p-p65 and p65. (**B**) The ratio of p-p65/p65. (**C**) The protein expression levels of p-IκBα and IκBα. (**D**) The ratio of p-IκBα/IκBα. GAPDH was utilized as an internal control. Data are presented as the mean ± SD (n = 3). Different letters indicate significant (*p* < 0.05) differences. C: the control group; Y: the Cy-treated group; LP: Cy + 200 mg/kg OP; HP: Cy + 400 mg/kg OP.

Western blotting was adopted to investigate the effect of OP on the intestinal NF-κB pathway of immunosuppressed mice induced by Cy. As shown in Figure 7, the NF-κB pathway of mice in the Y group was inhibited, and the phosphorylation levels of IκBα and p65 were significantly decreased (*p* < 0.05). OP can significantly activate the expression level of NF-κB pathway, increase the phosphorylation level of intestinal IκBα and p65, and alleviate the incidence of intestinal inflammation. The alleviating ability is enhanced with the increase in the concentration of OP. The results suggested that OP improved the immune capacity of immunosuppressed mice by regulating the NF-κB pathway.

### 2.7. Effects of OP on the Overall Structure of Gut Microbiota

**Figure 8:**
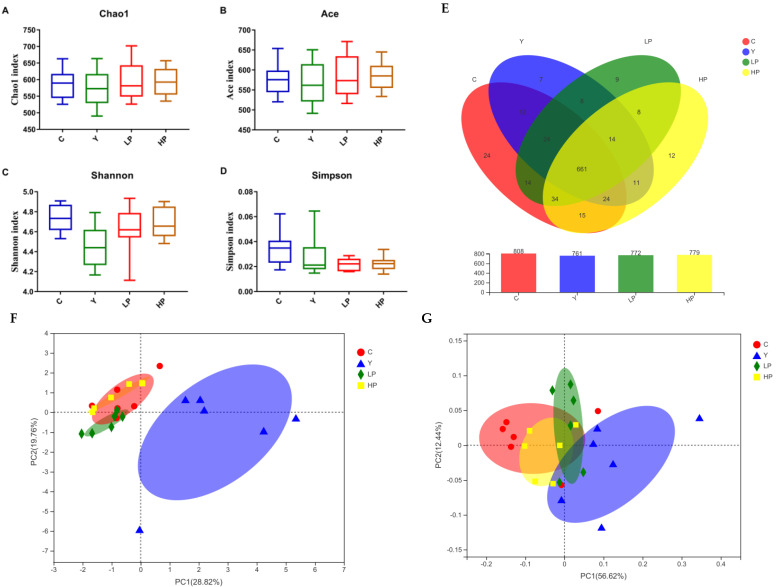
Effects of OP on the gut microbiota in the Cy-induced Immunosuppressed mice (n = 6 per group). (**A**) Chao1 index, (**B**) Ace index, (**C**) Shannon index, (**D**) Simpson index, (**E**) Venn diagram, (**F**) Principal components analysis (PCA), (**G**) Principal co-ordinates analysis (PCoA). C: the control group; Y: the Cy-treated group; LP: Cy + 200 mg/kg OP; HP: Cy + 400 mg/kg OP.

After mass filtering and splicing, a dataset consisting of 949824 Tags were spliced from a fecal sample of all the groups. The operational taxonomic units (OTUs) clustered with 97% similarity level was used for statistical analysis of biological information. The Venn diagram was used to compare the species and quantity of OTUs among different fractions, which could show the similarity and overlap of species composition more intuitively. As shown in Figure 8E, the numbers of OTUs in the C, Y, LP and HP groups were 808, 761, 772 and 779, respectively. The common OTU of the four groups was 661. The common number of OTUs shared between the C group and LP or HP groups was larger than those shared between Y and C groups. The specific OTUs in the Y group were significantly lower than that in the C group, while the number of specific OTUs was increased in the HP group.

Alpha diversity can reflect the abundance and diversity of the gut microbiome. The Chao1 index and Ace index are often employed to estimate the number of OTUs in the sample, while Shannon and Simpson indices were utilized to estimate the diversity of intestinal flora. In Figure 8A–D, the Chao and Ace index of the Y group was decreased compared with those of C, LP and HP groups, indicating that the community richness of the Y group was reduced by Cy, while the Shannon and Simpson indices of LP and HP groups were higher than Y group, indicating the higher community diversity in C, LP and HP groups. Under different environmental conditions, the species distribution of the intestinal flora community has certain similarities and differences. On the basis of the OTU abundance information and Bray–Curtis inter-sample distance matrix, principal component analysis (PCA) and principal co-ordinates analysis (PCoA) were utilized to compare the similarity or dissimilarity of sample colony composition, respectively. The β-diversity results in Figure 8F–G showed that the dispersion between the C group and Y group was relatively high, indicating a great difference in species composition, while the HP group showed great similarity to the C group in microbial flora structure. The above results indicated that the intestinal flora structure of Cy-induced immunosuppressed mice was changed due to survival pressure, while the intestinal flora of LP and HP groups was partially recovered, and the effect was more significant in the HP group.

### 2.8. Effects of OP on Microbial Changes

**Figure 9:**
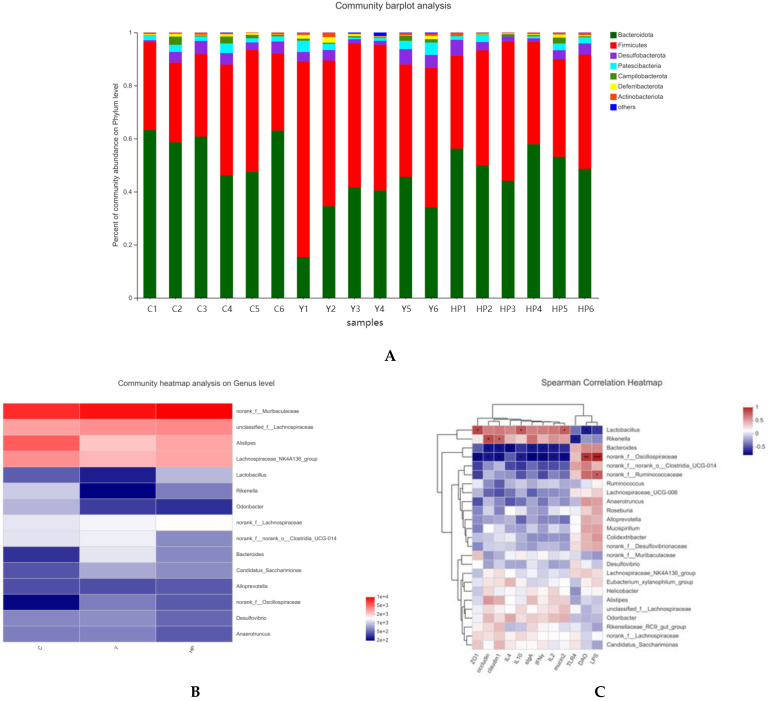
Effects of OP on community structure of intestinal microflora in immunosuppressed mice (n = 6 per group). (**A**) The community structures of gut microbiota at phylum levels. (**B**) The community structures of gut microbiota at genus levels. (**C**) Spearman’s correlations heatmap between bacterial taxa and host immune parameters. C: the control group; Y: the Cy-treated group; HP: Cy + 400 mg/kg OP. Different colors of squares represent different r values of Spearman’s correlation. (* *p* < 0.05; ** *p* < 0.01; *** *p* < 0.001).

In order to further investigate the specific changes induced by Cy and OP on intestinal flora, we analyzed the taxonomic composition of samples in various groups at the levels of phylum and genus. At the phylum level (Figure 9A), the gut microbiota was mainly composed of *Firmicutes, Bacteroidetes, Desulfobacterota, Patescibacteria, Campilobacterota, Deferribacterota* and *Actinobacteria*. Among them, *Firmicutes* and *Bacteroidetes* accounted for more than 95%. Compared with the C group, *Firmicutes* and *Desulfobacterota* were increased in the Y group, while *Bacteroidetes* was decreased. In contrast, treatment with OP partially reversed Cy-induced intestinal flora dysregulation, although not significantly. In order to further understand the changes in the microbial community, the species information of all detected samples at the level of the top 15 genera was drawn into a clustering heat map. At the genus level, as shown in Figure 9B, it can be seen that *norank_f_Muribaculaceae, Alistipes, lachnospiraceae_NK4A136_group, Rikenella, Lactobacillus, Bacteroides, Desulfovibrio* and *norank_f_Oscillospiraceae* were the most predominant microbiota. The abundance of *Desulfovibrio* was increased in the Y group and reversed by treatment with OP. The abundances of *Alistipes, Lactobacillus, Rikenella* and *lachnospiraceae_NK4A136_group* were decreased in the Cy-treated mice when compared with the healthy mice, while the abundance of *norank_f_Oscillospiraceae, Bacteroides, Candidatus_Saccharimonas, Anaerostipes* and *norank_f_norank_o_Clostridia_UCG_014* were increased after Cy treatment. The administration of OP could partially reverse these imbalances induced by Cy. Notably, OP supplementation could significantly promote the abundance of beneficial probiotic *Lactobacillus*, which plays a significant role in intestinal health.

Spearman’s correlation analysis was performed to further evaluate the effects of OP supplementation on intestinal microflora and immune response parameters. In Figure 9C, the results showed that the levels of *Lactobacillus* and *Rikenella* are negatively correlated with the secretion contents of DAO and LPS, positively correlated with the secretion of sIgA and the mRNA expression of ileum-related genes (IL-2, IFN-γ, IL-4, IL-10, ZO-1, Occludin, Claudin-1, mucin-2, TLR4). *Norank_f_Oscillospiraceae* showed a significantly positive correlation with LPS and DAO. Meanwhile, *norank_f_Oscillospiraceae* and *Bacteroides* were negatively correlated with all immune traits (IL-2, IFN-γ, IL-4, ZO-1, Occludin, Claudin-1, mucin-2, TLR4).

### 2.9. OP Promoted Production of SCFAs

**Figure 10:**
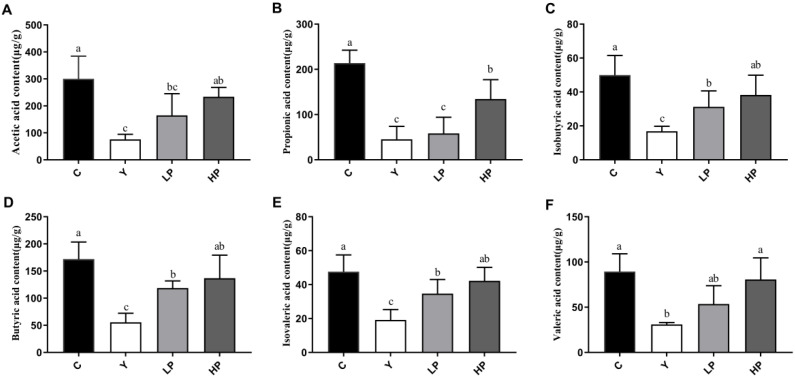
Effects of OP on the short-chain fatty acid content in immunosuppressed mice. (**A**) Acetic acid, (**B**) Propionic acid, (**C**) Isobutyric acid, (**D**) Butyric acid, (**E**) Isovaleric acid, (**F**) Valeric acid. All data are presented as the mean ± SD (n = 6). Different letters indicate significant (*p* < 0.05) differences. C: the control group; Y: the Cy-treated group; LP: Cy + 200 mg/kg OP; HP: Cy + 400 mg/kg OP.

**Figure 11:**
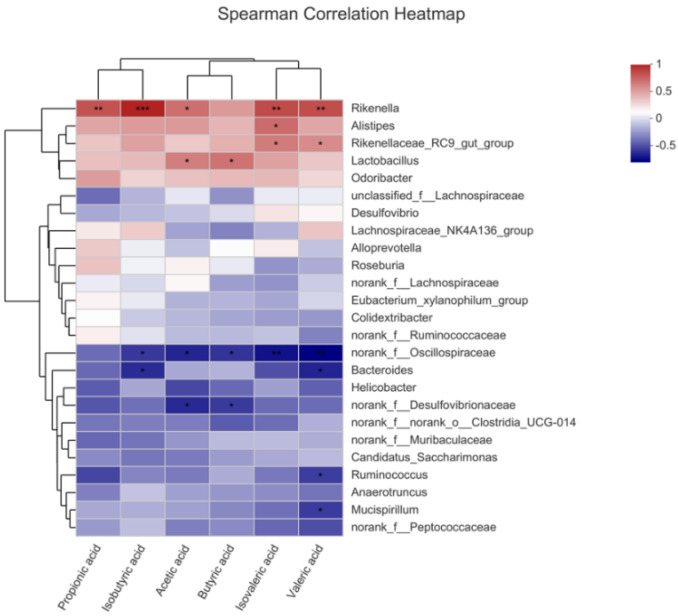
Spearman’s correlations heatmap between bacterial taxa and short-chain fatty acids (n = 6 per group). Different colors of squares represent different r values of Spearman’s correlation. (* *p* < 0.05; ** *p* < 0.01; *** *p* < 0.001).

SCFAs are key bacterial metabolites in the gut, which are beneficial to human health. In this study, a total of six kinds of SCFAs were detected from mice feces, consist of acetic acid, propionic acid, isobutyric acid, butyric acid, isovaleric acid and valeric acid. Compared with the C group, the contents of those SCFAs in the Y group were significantly reduced (Figure 10A–F), while the intake of OP reversed this decline. As shown in Figure 11, *Rikenella, Lactobacillus, Alistipes* and *Odoribacter* were positively correlated with all the SCFAs, while *norank_f_Oscillospiraceae* and *norank_f_Desulfovibrionaceae* were negatively correlated with most of the SCFAs, such as acetic acid, butyric acid and valeric acid. These results indicated that OP promoted the production of SCFAs, which was consistent with the increase in the abundance of SCFAs-producing microbiota.

## 3. Discussion

At present, among the various natural bioactive peptides with health-promoting properties, the search for molecules able to enhance immune response is one of the research hotspots [28,31]. The intestinal is the largest immune organ in the human body, which contains numerous immune cells. The gut mucosal immunity system is mainly composed of lymphocytes, macrophages and plasma cells, which is the first natural barrier against potential environmental damage [20,44]. Cy is a commonly used anti-tumor drug as well as an immunosuppressant, which may cause the imbalance of the body’s immune function [45]. Therefore, we selected the immunosuppressive animal model created by Cy to investigate the effects of OP on immune regulation. It is generally accepted that damage to the intestinal barrier induced by chemotherapeutic drugs will cause intestinal and systemic inflammation, diarrhoea, enteritis and systemic immune deficiency, leading to high mortality [46]. Maintaining intestinal mucosal immune function is of great significance to human health. After the intestinal mucosa contacts the antigen, the lymphatic tissues in the mucosa immediately produce an immune response and secrete immune globulin to prevent harmful antigens such as bacteria and viruses from invading the small intestine. Secretory immunoglobulin A (sIgA) is the immunoglobulin of the small intestinal mucosa, which is mainly secreted by mature plasma cells. sIgA plays a key role in maintaining intestinal homeostasis and keeping the balance of the immune system, which is the first line of defense against enterotoxins and pathogenic microorganisms [47]. Cy could reduce the content of sIgA in mice. Peptides from Alaska Pollock can promote the production of sIgA as reported by Li et al. before [48]. In this research, OP also significantly increased the secretion of sIgA.

The integrity and permeability of the intestinal barrier are important indicators of intestinal health. The levels of DAO and LPS in the intestine reflect the integrity and disruption of the gut mechanical barrier. Lipopolysaccharide (LPS) produced by the intestinal flora is released from the gut into the bloodstream, thereby increasing the inflammatory response. DAO is a kind of highly active intracellular enzyme in mammalian intestinal epithelial cells. Once the intestinal epithelial cells have been stripped and dissolved, the DAO would be released into the intestine and the blood stream, and DAO activity is closely related to the nucleic acid and protein synthesis of the mucosal cells. In this study, OP protected the integrity of tight junction and repaired intestinal permeability damage caused by LPS. Tight junction between intestinal cells is an important structural basis for intestinal barrier function, which effectively regulates gut permeability and maintains epithelial cell barrier. When the intestinal barrier function is healthy and normal, tight junction regulates cellular bypass by regulating the intercellular space between adjacent cells, allowing nutrients to enter the intestinal epithelium freely and preventing the passage of intestinal bacteria, toxins and inflammatory mediators, so as to maintain the integrity of the protective function of the intestinal mucosal barrier. Tight junction proteins, including claudin, occludin, zonula occludens (ZO) and a number of other proteins, could maintain the integrity of tight junctions. Transmembrane proteins (claudin and occludin), connect two adjacent cells through the interaction of the extracellular ring. Abnormal expression of Claudin-1 is a common characteristic of many illnesses, which reflects the injury of the intestinal barrier. The decreased expression and abnormal distribution of Occludin lead to increased permeability of intestinal epithelial cells [49]. ZO-1 connects Claudin and Occludin to the cytoskeleton, which is usually employed as an indicator of gut barrier function [50]. Mucin-2 is produced by goblet cells and also plays a critical role in maintaining the intestinal mucosal barrier function [51,52]. Cy damaged the intestinal mucosal barrier and decreased the mRNA expressions of intestinal tight junction proteins ZO-1, Occludin and Claudin-1, and OP significantly increased the gene levels of ZO-1, Occludin, Claudin-1 and Mucin-2, indicating that OP effectively restored intestinal barrier function. Polysaccharides from *Auricularia auricula* (AAP) alleviated intestinal mucosal injury by up-regulating the levels of ZO-1, Claudin-1 and Occludin in Cy-treated mice, which was consistent with our study [49].

The immune system consists of immune organs, immune cells and immune molecules, which resist attacks of the outside world and maintain human health [53]. When the immune function is suppressed, the body will inevitably be affected by diseases such as infection and cancer. The main immune organs include the thymus, spleen and liver. The spleen is not only the organ where immune cells settle, but also where they accept antigenic stimuli to generate immune responses. T lymphocytes differentiate and mature in the thymus, which participates in regulating the maturation of T cells in peripheral blood [54]. T cells play a significant role in a variety of immune responses against cancers and some pathogens. The increased weight of the spleen and thymus indicates the proliferation of lymphocytes, while the decrease in immunity may be due to the atrophy of the immune organs. The liver is a significant metabolism and immune organ, which is known as the “life tower”. The various metabolism, detoxification and immune functions of the body are undertaken by the liver. The liver is rich in cellular components, such as dendritic cells, stellate cells, Kupffer cells, sinus endothelial cells and lymphocytes, which secrete a series of cytokines and are involved in the immune regulation of the body. The liver also actively transfers sIgA to bile, which acts as a protective agent by delivering high levels of sIgA to the intestinal cavity [55]. The decrease in the index of immune organs indicates a decrease in immune function [56]. The results obtained in this study showed that the thymus, spleen and liver index were significantly increased. The conclusion was in accordance with the experimental results of Huang et al., indicating the index of immune organs was up-regulated by the intake of pulp polysaccharides [57]. Immune cells are mainly divided into B cells, T cells and NK cells. They regulate cell growth and differentiation through binding to corresponding cell surface receptors, thus modulating immune response [58]. Regulatory T cells act as an indispensable role in keeping immune adaptation to autoantigens and are involved in the regulation of immune responses to ameliorate the inflammatory process. Cytokines are small soluble extracellular peptides or glycoproteins that have a variety of physiological functions, mainly including interleukin, tumor necrosis factor and growth factors. Cytokines are important factors in mediating and regulating the immune response. Therefore, the secretion level of cytokines could represent the immune function of the body. Th1 and Th2 secrete various types of cytokines to regulate immunity, leading to a variety of cellular responses [59]. IL-2 and IFN-γ are typical cytokines of Th1, while IL-4 and IL-10 are typical cytokines of Th2 [60]. IL-2 promotes the growth and differentiation of T cells, induce the differentiation of killer cells and stimulate the immune response [54]. IFN-γ affects the levels of tight junction proteins, and causes endocytosis of occludin and claudin-1. IFN-γ can promote T cell differentiation and enhance antimicrobial immunity in macrophages as well [61]. Produced by Th2 cells, IL-4 regulates allergic reactions, activates immune response against parasites, and is also a key cytokine to activate goblet cells and promote the production of immunoglobulin [49]. IL-10 promotes immune regulation and controls inflammation in the body, which is mainly derived from macrophages in nomal tissues [62]. Cy will cause intestinal cellular immune dysfunction, resulting in Th1/Th2 imbalance [13]. Polysaccharides from *Malus halliana Koehne* flowers significantly boosted the content of IL-2, TNF-α, and IFN-γ and gene expression of these cytokines, and improved the humoral and cellular immune responses in Cy-induced immunosuppression model [53]. Compared with mice treated with Cy, peptides from *Nibea japonica* muscles significantly up-regulated the secretion of cytokine, such as IL-2, IFN-γ and TNF-α [63]. In this study, OP markedly up-regulated the contents of IFN-γ, IL-2, IL-4 and IL-10 and the gene expressions in immunosuppressed mice, suggesting that OP treatment could mediate Th1/Th2 balance.

Studies have shown that TLR4 could recognize bioactive substances and transduce intracellular signals via the downstream signaling molecule MyD88, which releases TNF-α, IL-1β and other cytokines in the host innate immune response through activating the NF-κB signaling pathway [64,65]. Nuclear factor-κB (NF-κB) is a pivotal transcription factor that regulates immune and inflammatory responses. It is usually active in the form of p50-p65 heterodimer and its inhibitory protein IκB [52]. NF-κB is composed of the dimeric proteins p65 and p50 of the Rel family that control genes for many biological processes, including innate and acquired immunity, inflammation, lymphatic organ production, B cell formation, and stress response [66]. The activation of NF-κB is related to the genes encoding pro-inflammatory cytokines, which participate in various biological processes such as immune response, inflammatory response, cell apoptosis, and tumorigenesis by regulating the expression of various genes [67]. Cy can damage intestinal mucosa, cause intestinal damage, affect the secretion of intestinal cytokines, and regulate TLRs, NF-κB and other signaling pathways. A research study found that peptides from *Solenocera crassicornis* have immunomodulatory activity through the NF-κB signaling pathway, and have an important protective effect on immunosuppressed mouse models [68]. Glycosaminoglycan from *Apostichopus japonicus* [66] and *Cordyceps sinensis* [14] polysaccharides can balance the imbalance of cytokine secretion, regulate the NF-κB pathway, and repair Cy-induced intestinal immune dysfunction in mice. In this study, OP significantly enhanced the expression of p65 and IκBα phosphorylated proteins, suggesting that OP may play a protective role by activating the NF-κB pathway.

The intestinal flora plays a vital role in maintaining intestinal health. Probiotics and opportunistic pathogens are in a state of dynamic balance, once the balance is disturbed, it is easy to cause infection [69]. In addition, intestinal flora also regulates the immune function of T and B cells, maintains the immune balance and coordinates the immune tolerance of the body [70]. Community richness mainly included Chao 1 and Ace index, and community diversity mainly included Shannon and Simpson index. The index of Chao1 and Ace can estimate the number of OTU contained in the sample. The higher the value is, the higher the richness of intestinal flora [71]. Simpson and Shannon index can be utilized to estimate microbial diversity in the sample. The diversity of intestinal microbiota composition is beneficial to the self-regulation of intestinal microorganisms. In this study, α-diversity and β-diversity analysis showed that the richness index, diversity index and PCA analysis results of immunosuppressed mice treated with OP were similar to those of control mice, indicating that OP reversed the changes of intestinal microflora in mice induced by Cy. The gut microbial composition of mice was studied at the OTU taxonomic level. The results showed that the total OTUs in the Cy group significantly decreased, while the number of OTUs in the OP group was closer to that in the control group, which may be due to the fact that OP treatment increased the amount of beneficial bacteria until it returned to a normal level. In our study, at the phylum level, compared with the control group, the abundance of *Firmicutes* in Cy group rose, while the abundance of *Bacteroidetes* decreased. The increase in *Firmicutes*/*Bacteroidetes* ratio was consistent with previous studies [5,72]. These results suggested that increasing the ratio of *Firmicutes*/*Bacteroidetes* may promote immunosuppression. *Firmicutes* and *Bacteroidetes* are the two most common types of bacteria in the gut. *Firmicutes* participate in host metabolism primarily through the synthesis of SCFAs [73]. *Bacteroidetes* is the cornerstone of a healthy intestinal environment, which is involved in immune regulation. At the genus level, *Desulfovibrio, Norank_f_oscillospiraceae, Bacteroides, Candidatus_saccharimonas, Anaerostipes* and *Norank_F_Norank_O_Clostridia_UCG_014* had high abundance in the Cy group, which might be pathogenic. However, the OP showed a reversal to the normal state after treatment. LPS was mainly secreted by *Bacteroidetes* and *Desulfovibrio*, which was recognized as a stimulator of inflammation [70]. *Desulfovibrio* is one of the main members of *Proteobacteria*, which could produce hydrogen sulfide to destroy the intestinal mucosal barrier and might cause intestinal inflammation [74,75]. *Bacteroidetes* are the dominant bacteria in the intestinal tract. When the immunity is low, it is easy to cause endogenous infection, which can damage the health of the host. Moreover, the relative abundance of *Alistipes*, *Lactobacillus, Rikenella* and *Lachnospiraceae_NK4A136_group* in Cy-treated mice increased after OP treatment. *Alistipes* and *Odoribacter* could help reduce intestinal inflammation and promote intestinal maturation [76]. Probiotics are generally considered to be living microbial additives that offer health benefits to the host by maintaining the balance of gut microbiota. *Lactobacillus* is the most common probiotic genus which is important for maintaining healthy homeostasis [77]. *Lactobacillus*, as the dominant bacteria in the intestinal tract, can promote the expression and reasonable distribution of tight junction proteins in epithelial cells, and maintain the barrier function of epithelial cells [78]. At the same time, it can relieve the damage of pathogens and inflammatory cytokines induced on tight junctions and maintain the integrity of mucosa [60]. *Lactobacillus* also produces lactic acid, which acts as a precursor for butyric acid synthesis [79]. Previous research found that the relative abundance of *Lactobacillus* was increased after the treatment of *Chrysanthemum morifolium* polysaccharide, which had immune protection effects on immunosuppressed mouse intestine [73].

SCFAs, including acetic acid, propionic acid, butyric acid and valeric acid, are the main metabolites secreted by intestinal microorganisms and could maintain the normal function of the immune system [21]. SCFAs secreted by gut microflora might inhibit the production of inflammatory cytokines and promote the content of sIgA [80]. In this research, compared with the Cy group, the concentrations of SCFAs in mice feces were observably increased after OP treatment, indicating that intestinal microbial activity was increased. It has recently been reported that butyrate maintained intestinal homeostasis through anti-inflammatory effects, which had specific immunomodulatory functions and enhanced the overall integrity of the intestinal barrier [20,81]. Propionate was the main microbial fermentation metabolite in the intestinal tract, which provided energy for intestinal epithelial cells, promoted the differentiation of T cells and played an inhibitory role in the occurrence of disease [56,82]. *Rikenella* was SCFA-producing bacteria, which fermented propionate to produce energy and promoted gluconeogenesis [83]. Several studies have proved that SCFAs could not only maintain intestinal homeostasis, but also improve the immune level [84]. Some bioactive substances, such as sulfated modification yam polysaccharide [85] and *Nigella sativa* seed polysaccharide [56], exerted their immunomodulatory activity by restoring the generation of SCFAs. In conclusion, we found that OP improved intestinal flora composition and significantly enhanced the production of SCFAs, suggesting that OP may play an immune protective role in immunosuppressed mice.

## 4. Materials and Methods

### 4.1. Materials and Reagents

The fresh oysters (*Crassostrea gigas*) were sampled from the Yellow Sea, China. Oyster peptides were extracted and prepared according to our previous report [86]. Cy was purchased from Sigma-Aldrich (St. Louis, MO, USA). Secretory immunoglobulin A (sIgA) and Lipopolysaccharides (LPS) Enzyme-Linked Immunosorbent Assay (ELISA) kits were bought from Huamei Bio-engineering Co., Ltd. (Wuhan, China). Diamine oxidase (DAO) ELISA kit was obtained from Nanjing SenBeiJia Biological Technology Co., Ltd. (Nanjing, China). IFN-γ, IL-2, IL-4 and IL-10 Enzyme-Linked Immunosorbent Assay (ELISA) kits were purchased from Boshide Bioengineering Limited Company (Wuhan, China). All other chemical reagents used in this study were of analytical grade or the highest grade.

### 4.2. Animals and Experimental Design

A total number of 32 male-specific pathogen-free (SPF) BALB/c mice, aged 5-6 weeks old (weight 15–18 g), were purchased from Shanghai Slaccas Laboratory Animal Company (Certificate Number SCXK (hu) 2017-0005, Shanghai, China). Before the experiment, the animals were acclimated to a new rodent facility for one week. The mice were fed in the rodent facility at 25 ± 1 °C with a relative humidity of 50 ± 3%, and 12 h light-darkness cycles were adopted for acclimatization. The water and standard mouse chow were provided for the mice ad libitum regularly. All animal testing procedures involved in this research were ratified by the Animal Ethics Committee of Zhejiang University of Technology (20210308038).

The experimental mice were randomly divided into four groups (n = 8) as follows: the control group (C), the cyclophosphamide group (Y), the high-dose and low-dose OP groups (HP and LP). The HP and LP groups were daily given 400 and 200 mg/kg OP gavage for 21 days, respectively. The mice in the C group and Y group were gavaged with an equal volume of normal saline. The Y group, HP group, and LP group were intraperitoneally injected with 50 mg/kg Cy on days 18, 19, 20, and 21, while mice in the C group were injected with normal saline in the same way. At the end of the administration period, after fasted for 12 h with free access to water, all the mice were weighed and euthanized. The thymus, spleen, liver, ileum, serum and fecal samples were collected immediately after execution, and stored at −80 °C for further analysis. The details of animal experiments were shown in Table 1.

### 4.3. Histopathologic Analysis

Each mouse ileum was excised and fixed with 2.5% (v/v) glutaraldehyde at 4 °C. Scanning electron microscopy (SEM) and transmission electron microscopy (TEM) were used to treat the samples. TEM samples were washed with 0.1 M PBS 3 times, fixed with 1% osmium acid for an hour, and washed with distilled water. The sample was then treated with gradient ethanol, dehydrated with pure acetone, embedded overnight in a Spurr resin and polymerized at 70 °C. The ileum was cut into thin sections and stained with uranium acetate and lead citrate for TEM. SEM samples were first dehydrated with gradient ethanol, then dried at the critical point of liquid CO_2_, and then plated with gold about 30 nm thick on the ileum surface by ion sputtering apparatus, and observed by scanning electron microscope. Zhejiang Academy of Agricultural Sciences was commissioned to handle the experiment.

### 4.4. Analysis of DAO and LPS in Serum

The mice were anesthetized and sacrificed. The whole blood samples collected were centrifuged at 4 °C for 10 min with 4000 r/min. The serum was obtained. Serum levels of DAO and LPS were assessed by following the manufacturer’s instructions.

### 4.5. Intestinal Cytokines

In each group, 100 mg of ileum was weighed, and 1 mL of sterile normal saline was added. The ileum samples were homogenized to mucus by electric homogenizer, and put into a precooled centrifuge at 4 °C, centrifuged at the condition of 4000 r/min for 10 min. The supernatant was then collected and stored in a refrigerator at −80 °C. The contents of sIgA, IFN-γ, IL-2, IL-4 and IL-10 in the ileum homogenate were determined by using ELISA kits, according to kit instructions.

### 4.6. Intestine mRNA Analyses

The relative mRNA expressions of IFN-γ, IL-2, IL-4, IL-10, TLR4, Mucin-2, Occludin and claudin-1 were detected by real-time quantitative polymerase chain reaction (RT-qPCR). The β-actin was used as the reference gene for normalization. The primers used in this study were synthesized through Sangon Biotech (Shanghai, China) Co., Ltd., which are shown in Table 2. In brief, total RNA from ileum tissue was extracted by RNAiso Plus (Takara, Dalian, China), according to the manufacturer’s instructions. The extracted RNA purity and concentration were determined using a NanoPhotometer (P300, Implen, Germany). The Transcriptor First Strand cDNA Synthesis Kit RNA (Roche, Germany) was adopted for reverse transcription of RNA and the synthesized cDNA were temporarily stored at −20°C. The PCR amplification reactions were performed on the Bio-rad CFX96 real-time PCR System (Bio-rad, Foster City, CA, USA). The relative gene levels were calculated by the 2^−^^△△Ct^ method. 

### 4.7. Western Blot Analysis

Western blot analysis was performed by Fan et al. method [27]. Briefly, 100 mg of ileum tissue was added to a lysate buffer containing 1 mL pre-cooled radioimmunoprecipitation assay (RIPA) lysis buffer, 10 μL 50-mM phenyl methyl sulfonyl fluorides (PMSF) and 10 μL protease inhibitor and homogenized. The mixture was lysed at 4 °C for 15 min and then centrifuged for 10 min at 12,000 r/min. The supernatant protein concentration was evaluated according to the BCA protein assay kit (Beyotime, Shanghai, China) instructions. The protein was denatured by boiling anionic denaturing detergent sodium dodecyl sulfate in the loading buffer solution at 100 °C for 5 min. The method of sodium dodecyl sulfate polyacrylamide gel electrophoresis (SDS-PAGE) was adapted to sever the denatured proteins and then transferred onto a 0.45 µm polyvinylidenefluoride membrane. After blocking nonspecific antibodies by using 5% non-fat milk, membranes were hatched with proportionally diluted antibodies against p65, p-p65, IκBα, p-IκBα and GAPDH at 4 °C overnight. Washing with TBST three times, membranes were incubated with secondary antibody (HRP-conjugated secondary antibody) for an hour at room temperature. Proteins were detected by chemiluminescent imaging with an enhanced chemilu-minescence (ECL) reagent kit (Clinx, Shanghai, China). The relative densities were quantified using the ImageJ software (1.52 n, National Institutes of Health, Bethesda, MD, USA) and each test was carried out three times.

### 4.8. Fecal Microbiota Analysis

The cecal contents collected were promptly frozen by liquid nitrogen and stored at the temperature of −80 °C. Fecal microflora DNA from each fecal sample was extracted by using KAPA HiFi Hotstart PCR Kit (KAPA Biosystems, Boston, MA, USA) following the manufacturer’s instructions. PCR amplification of the V3-V4 region of bacterial 16 S rRNA was carried out with forward primer 338F (ACTCCTACGGGAGCAGCAG) and reverse primer 806R (GGACTACHVGGGTWTCTAAT), and sequenced through the Illumina MiSeq platform (Illumina, SD, USA) of Hangzhou HanTai Gene Co., Ltd. (Hangzhou, China). Flash software (V1.2.11) (Baltimore, MD, USA) was used to match the raw data, and the Quality Control software package was used for filtering. A sequences clustering was performed using Parse (v.7.1) on the basis of RDP Classifier (v.2.11) to divide into operational taxonomic units (OTUs) with 3% divergence. The processing of data was performed through the Majorbio Cloud Platform.

### 4.9. Quantification of SCFAs in Cecum Content

The content of SCFAs in cecum was measured according to Chen et al. [64], with minor modifications. The cecal contents of mice in each group were weighed and 100 mg were dissolved in distilled water at the ratio of 1:5 (*w*/*v*). After centrifuging at 8000 r/min for 5 min, the supernatant was gathered and mingled with 2-ethylbutyric acid (25 mM internal standard) by an equal volume. The supernatant was centrifuged once more and added into 0.15 M oxalic acid solution. The supernatant obtained by centrifugation was used for gas chromatography (GC) analysis. The Agilent 7890B gas chromatograph (GC) system equipped with a flame ionization detector (FID) and an HP-INNO WAX column (15 m × 0.25 mm × 0.25 μm) was applied for analysis (Agilent Technologies Inc., Santa Clara, CA, USA). The temperatures of the FID and injection were 250 °C and 230 °C, respectively. The hydrogen was employed as carrier gas, the flow velocity was 30.0 mL/min, and the split ratio was maintained between 1 and 10. The injection volume was 1 μL and run for 22 min each time. The standard curve was created using the acetic acid, propionic acid, n-butyric acid, isobutyric acid, n-valeric acid and isovaleric acid (Aladdin Reagent Co. Ltd., Shanghai, China).

### 4.10. Statistical Analysis

SPSS 17.0 software (SPSS Inc., Chicago, IL, USA) was used for statistical analysis. The statistical comparisons were performed by one-way ANOVA. All values were reported as mean ± standard deviation (SD) and different letters were used to indicate significant differences. The Base Calling was used to convert high-throughput sequencing image data into sequences and Spearman’s correlation analysis was adopted to evaluate the correlation coefficients between intestinal flora and immune-related traits.

## 5. Conclusions

In this study, OP showed immune-protective effects on intestinal immunosuppression in Cy-treated mice. OP significantly increased the index of immune organs, restored the integrity of intestinal mucosal, stimulated the secretion of cytokines (IL-2, IFN-γ, IL-4 and IL-10) and sIgA in serum, and increased the mRNA expression of Claudin-1, Occludin and ZO-1. OP also alleviated Cy-induced intestinal injury via activating the NF-κB pathway. Moreover, OP regulated the composition of intestinal microorganisms in immunosuppressive mice and enhanced the synthesis of SCFAs. Our results suggest that OP can be utilized as a potentially health-promoting regulator of the gut microbiota or an immunomodulator in foods or drugs.

## Figures and Tables

**Table 1:** The grouping scheme of experimental mice.

Groups	Oral Administration (Days 1–21)	Intraperitoneal Injection (Days 18–21)
C	Saline	Saline
Y	Saline	50 mg Cy /kg BW/day
LP	200 mg OP/kg BW/day	50 mg Cy /kg BW/day
HP	400 mg OP/kg BW/day	50 mg Cy /kg BW/day

**Table 2:** Primers of RT-PCR.

Gene	Gene Accession Number	Primer Sequence 5′-3′	Product Size(bp)
IFN-γ	NM_008337.4	F: CGGCACAGTCATTGAAAGCC R: TGTCACCATCCTTTTGCCAGT	119
IL-2	NM_008366.3	F: CTCTGCGGCATGTTCTGGAT R: AATGTGTTGTCAGAGCCCTTT	118
IL-4	NM_021283.2	F: CCATATCCACGGATGCGACA R: CTGTGGTGTTCTTCGTTGCTG	131
IL-10	NM_010548.2	F: GGTTGCCAAGCCTTATCGGA R: GAGAAATCGATGACAGCGCC	156
TLR4	NM_021297.3	F: TTGAATCCCTGCATAGAGGTAG R: TTCAAGGGGTTGAAGCTCAGAT	125
Mucin-2	NM_023566.3	F: CCGGATCTATGCCGTTGCTA R: TCCAGGTGGGTATCGAGTGT	126
Occludin	NM_001360539.1	F: TAGGGGCTCGGCAGGCTAT R: CCGATCCATCTTTCTTCGGGT	104
Claudin-1	NM_016674.4	F: CAACCCGAGCCTTGATGGTA R: ACTAATGTCGCCAGACCTGAAA	169
β-actin	NM_007393.5	F: TATAAAACCCGGCGGCGCA R: TCATCCATGGCGAACTGGTG	117

F, forward; R, reverse.

## Data Availability

The data presented in this study are available on request from the corresponding author. The data are not publicly available due to public availability violating the consent that was given by research participants.
